# Evaluating red blood cell distribution width from community blood tests as a predictor of hospitalization and mortality in adults with SARS-CoV-2: a cohort study

**DOI:** 10.1080/07853890.2021.1968484

**Published:** 2021-08-19

**Authors:** Tamar Banon, Joshua Wortsman, Shay Ben Moshe, Sivan Gazit, Asaf Peretz, Amir Ben Tov, Gabriel Chodick, Galit Perez, Tal Patalon

**Affiliations:** a Maccabitech Institute for Research & Innovation, Maccabi Healthcare Services, Tel Aviv, Israel; b Kahn Sagol Maccabi Research & Innovation Center, Maccabi Healthcare Services, Tel Aviv, Israel; c Department of Computer Science, Ben-Gurion University, Beer Sheva, Israel; d Head Internal Medicine COVID-19 Ward, Samson Assuta Ashdod University Hospital, Ashdod, Israel; e Sackler Faculty of Medicine, Tel Aviv University, Tel Aviv, Israel

**Keywords:** COVID-19, SARS-CoV-2, RDW

## Abstract

**Background:**

Red blood cell distribution width (RDW) has been assessed during COVID-19 patient hospitalization, however, further research should be done to evaluate RDW from routine community blood tests, before infection, as a risk factor for COVID-19 related hospitalization and mortality.

**Patients and methods:**

RDW was measured as a predictor along with age, sex, chronic illnesses, and BMI in logistic regressions to predict hospitalization and mortality. Hospitalization and mortality odds ratios (ORs) were estimated with 95% confidence intervals (CI). RDW was evaluated separately as continuous and discrete (High RDW ≥ 14.5) variables.

**Results:**

Four thousand one hundred and sixty-eight patients were included in this study, where 824 patients (19.8%) had a high RDW value ≥14.5% (High RDW: 64.7% were female, mean age 58 years [±22] *vs.* Normal RDW: 60.2% female, mean age 46 years [±19]). Eight hundred and twenty-nine patients had a hospitalization, where the median time between positive PCR and hospital entry was 5 [IQR 1–18] days. Models were analyzed with RDW (continuous) and adjusted for age, sex, comorbidities, and BMI suggested an OR of 1.242 [95% CI = 1.187–2.688] for hospitalization and an OR of 2.911 [95% CI = 1.928–4.395] for mortality (*p* < .001). RDW (discrete) with the same adjustments presented an OR of 2.232 [95% CI = 1.853–1.300] for hospitalization and an OR of 1.263 [95% CI = 1.166–1.368] for mortality (*p* < .001).

**Conclusions:**

High RDW values obtained from community blood tests are associated with greater odds of hospitalization and mortality for patients with COVID-19.

**KEY MESSAGES**
- RDW measures before SARS-CoV-2 infection is a predictive factor for hospitalization and mortality.
- RDW threshold of 14.5% provides high sensitivity and specificity for COVID-19 related mortality, comparatively to other blood tests.
- Patient records should be accessed by clinicians for prior RDW results, if available, followed by further monitoring.

## Introduction

Coronavirus Disease 2019 (COVID-19), caused by the severe acute respiratory syndrome corona virus-2 (SARS-CoV-2), is the respiratory illness responsible for the global pandemic that has led to millions of deaths worldwide and caused widespread economic damage. To better mitigate the effects of the virus and improve medical outcomes, it is imperative to identify clinical, demographic, and laboratory factors predictive of clinical deterioration and prognosis of patients affected with this virus [1].

Blood tests are affordable, fast, and minimally invasive prognostic indicators that have proven useful in assessing COVID-19 disease progression. Previous research analyzing laboratory results in COVID-19 patients has demonstrated that they have more lymphopenia, thrombocytopenia, and overall leukopoenia at hospital admission [2]. Patients admitted to the ICU have an associated increased D-dimer and further decreased lymphocyte count [3]. Additionally, a high red blood cell distribution width (RDW) at the time of hospital admission, and increasingly elevated RDW during the hospital stay, have been associated with greater morbidity and mortality of COVID-19 patients [4,5].

RDW is a routine blood measure taken as part of a complete blood count and it quantifies anisocytosis by displaying the variation of red blood cell count (RBC) volumes [6]. The RDW measures size variance between RBCs and throughout an individual RBC’s ∼115-day lifespan [7,8]. It has gained greater attention in the last years due to its demonstrated ability to predict the risk of death in the general population, specifically in patients with non-cardiovascular critical illness, sepsis, pneumonia, and other respiratory tract infections [9–12]. Although the exact mechanism of how these disease processes increase RDW values is not entirely understood, it is postulated that pro-inflammatory states lead to insufficient and delayed erythropoiesis with structural and functional alteration of RBCs [9] as well as increased production and turnover of leukocytes and platelets [13,14].

In the year 2020, multiple studies demonstrated that RDW is a significant and independently powerful prognostic indicator for hospitalized COVID-19 patients [4,5]. For instance, Brody et al., demonstrated that COVID-19 patients hospitalized with an RDW value above the upper limits of normal have a significantly greater risk of mortality compared to those hospitalized with an RDW value within normal limits. Recent research also demonstrated that in-hospital measurements of RDW can be used as a univariate prognostic indicator that remains significant when adjusted for age, sex, comorbidities, and other blood measures [1,4].

However, the predictive value of RDW measurements obtained in the community before an individual diagnosis of COVID-19 is an uninvestigated topic of potential prognostic value. If RDW can act as a predictive measure to understand potential disease progression at the time of COVID-19 diagnosis, then this would allow for risk stratification and resource allocation to both observe and treat patients who are at greater risk for a more severe disease course [4,5].

In this study, the main objective is to determine if community-based RDW values obtained shortly before COVID-19 diagnosis are associated with increased hospitalization and mortality for COVID-19 patients.

## Methods

### Data source

This study was conducted using data from the second largest state-mandated healthcare provider in Israel, Maccabi Healthcare Services (MHS). The MHS central computerized database contains more than 2.5 million members (over 25% of the population) and is considered a representative sample of the population in Israel. The database captures all information on patient interactions within the healthcare system, which includes demographics, hospitalizations (inpatient and outpatient visits), disease diagnoses, prescriptions, and procedures. RDW and other laboratory measurement data were collected from community blood tests from various MHS clinics.

### Study population and design

This retrospective cohort study included all MHS patients that tested positive for SARS-CoV-2 infection between March 12, 2020 and January 24, 2021. Additionally, only patients with a recently recorded RDW test result, up to 14 days before COVID-19 diagnosis, were included in this research (up to February 7, 2021). Patients were diagnosed as COVID-19 positive based on a positive polymerase chain reaction (PCR) test in the community.

Hospitalization and mortality outcomes for this cohort were analyzed during a follow-up period beginning at the index date (positive COVID-19 PCR test) and ending on February 21, 2021.

### Study variables and definitions

The cohort was assessed at baseline (before their positive PCR test date [index date]), where patients were grouped as “Normal RDW” or “High RDW”. The recommended upper limit for RDW in the MHS database differs between 14% and 15.7%. In this study, High RDW was defined as ≥14.5%. Furthermore, MHS physicians refer to and follow *UpToDate*, which indicates that the normal RDW range is between 11.5 and 14.5% [15,16].

The complete blood count (CBC) included other blood tests, in addition to RDW, that were also assessed in this study. All CBC lab parameters were obtained from the same blood test on the same test date before patients' positive PCR result (more details in Statistical Analyses).

Variables evaluated at baseline include age, sex, socioeconomic status (SES), chronic diseases, and Body Mass Index (BMI). The SES is calculated based on the patients' residential area and presented from low to high with a ranking system (1 as the lowest and 10 as the highest). The system was built by Points Location Intelligence for commercial use, where geographic information systems and other relevant data (retail chains, credit cards, and housing) were used to develop a rank and score that is highly correlated with SES measured by the Israel Central Bureau of Statistics [17]. Three categories were used to define the SES variable in this study: low (1–4), medium (5–6), and high (7–10).

The MHS database has several automated chronic disease registries that were used to assess patient comorbidities. MHS uses artificial intelligence (AI) algorithms for detecting diagnoses and medication purchases to include patients in each specific registry, where the registries undergo continuous daily updates. The comorbidities assessed in this study include congestive health failure (CHF) [18], chronic kidney disease (CKD) [19], Diabetes [20], and Inflammatory bowel diseases (IBD) [21]. Cancer history was obtained from the National Cancer Registry [22]. Chronic obstructive pulmonary disease (COPD) patients were defined by an algorithm with several inclusions, where the main indicative criteria are the medication purchases (for COPD treatment) and diagnoses (International classification of diseases, ninth revision [ICD-9]) from a specialized MHS physician. All patients with a registry entry date before their positive PCR test were included in each comorbidity group. BMI was categorized using standard cut-points (underweight [BMI < 18.5], normal weight [BMI = 18.5–25], overweight [BMI = 25–30], or obese [BMI > 30]) [23]. Patients with missing SES and missing BMI were categorized as “Missing” in each variable (<4.0% of patients in each “missing” category).

Hospitalization and mortality outcomes were coded as binary variables, where a record of hospital entry date and/or death date was used to code whether these events occurred during the follow-up period. In this study, only COVID-19-related hospitalizations were considered, therefore only hospital data from the MHS COVID-19 registry were evaluated. Patients hospitalized for COVID-19 treatment are included in the MHS registry and require a record of a positive PCR test result and treatment in a COVID-19 specialty ward. This also includes patients who were hospitalized previously and then transferred to a COIVD-19 specialty ward, which may skew the average time from PCR to COVID-19 hospitalization for some.

### Statistical analyses

Descriptive statistics were presented as frequencies or mean values (standard deviation [±*SD*]) at baseline between Normal and High RDW patients. Days between RDW result and index and days between index and hospitalization, for patients who were hospitalized, were presented as mean (±*SD*) and median (interquartile range [IQR]). To assess statistical differences between groups, Mann–Whitney's test was used to evaluate age and Pearson's Chi-Squared test for discrete variables, such as sex, SES, chronic diseases, and BMI.

Receiver operating characteristic (ROC) curves were applied to assess the performance of several bloodwork laboratory tests, namely RDW, haemoglobin, lymphocytes, mean cell haemoglobin (MCH), mean cell volume (MCV), neutrophils, platelets, RBC, and white blood cell count (WBC). These tests were used to evaluate the sensitivity and specificity (diagnostic accuracy) for COVID-19 related hospitalization and mortality.

To assess the main objective, binary logistic regression models were implemented to evaluate two separate outcomes: hospitalization and mortality. The models in this study were multivariate analyses to predict the odds of hospitalization and mortality. Odds Ratios (OR) were evaluated for the independent variables (predictors) entered in each model. RDW was entered first as a continuous variable, and then again in a separate model as a discrete variable (Normal and High RDW [≥14.5%]). In the first step of each model, only the baseline RDW (continuous or discrete) was entered with age and sex (females compared to males). As a second step for each model, comorbidities were entered as binary covariates and BMI was entered with the Normal category defined as the comparison group. The second step models, including all covariates to reduce the possible bias of predictions, the *p*-value, ORs, and 95% confidence intervals (CI) were presented in tables. Additionally, Kaplan Meier survival curves (time from positive PCR to mortality) with risk tables are also performed and are presented in the Supplementary Materials.

A sub-analysis was also performed with patients under 50 years old without any comorbidities. Recent COVID-19 studies have stratified ages similarly, according to a systemic review, where patients over 50 years old and patients with various comorbidities had an increased risk of COVID-19 mortality [24]. Therefore, the objective of this sub-analysis was to evaluate RDW as a predictor in a healthy lower-risk population. Logistic regression was used to evaluate hospitalization outcomes, where covariates in this model included RDW, age, and sex. Mortality was not evaluated as there were no outcomes in this healthy cohort, which is similar to findings from a recent report on low COVID-19 case-fatality rates for persons under 50 years old [25].

## Results

**Figure 1.**
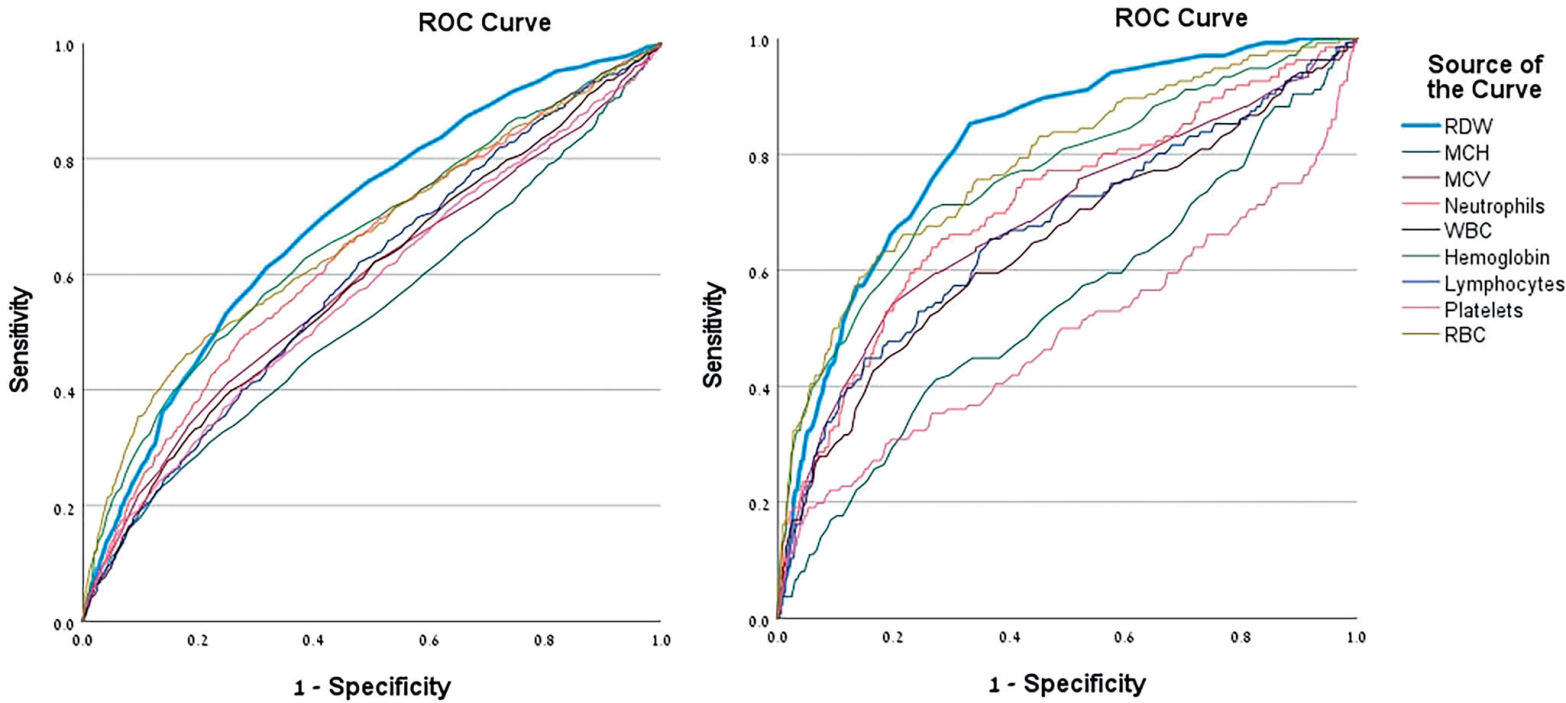
ROC curve with lab results as diagnostic predictors for hospitalization (left) and mortality (right).

A total of 4,168 adult patients (≥18 years old) were included in this study. Among them, 824 patients (19.8%) had a baseline RDW value greater or equal to 14.5% and were considered as High RDW patients for the remainder of this study. The mean RDW lab result at baseline for the full cohort was 13.6% (±1.7) and the median time from a blood test to positive PCR result was 6.7 [IQR 3.6, 10.6] days. The bloodwork lab results were evaluated with a ROC curve to illustrate the diagnostic ability of two binary variables, hospitalization (ROC area under the curve [AUC] for RDW = 0.692) mortality (AUC for RDW = 0.816). A 14.5% RDW threshold for hospitalization showed 36.6% sensitivity and 82.6% specificity, whereas a 14.5% threshold for mortality had 63.8% sensitivity and 81.7% specificity (Figure 1). Each laboratory optimal cut-point for COVID-19 related hospitalization and mortality was also constructed and presented in Supplementary Table 1. An RDW value of 13.6% had 61.4% sensitivity and 68.0% specificity for hospitalization and 86.2% sensitivity and 63.8% specificity for mortality. Additionally, the optimal cut-point for RDW was 13.75 (55.5% sensitivity and 68.0% specificity) and 14.35 (66.7% sensitivity and 80.4% specificity) for hospitalization and mortality outcomes, respectively.

**Table 1.** Baseline characteristic for COVID-19 patients with an RDW value up to two weeks before positive PCR test (*n* = 4168).

Baseline characteristics	Normal RDW (*n* = 3344)		High RDW (*n* = 824)		*p*-Value
	Patient count	Percentage (%)	Patient count	Percentage (%)	
Age (mean [±*SD*])	46 (19)		58 (22)		<.001
Sex					
Male	1332	39.8	291	35.3	.017
Female	2012	60.2	533	64.7	
SES					
Low (1–4)	1215	36.3	349	42.4	<.001
Med (5–6)	1044	31.2	281	34.1	
High (7–10)	1029	30.8	185	22.5	
Missing	56	1.7	9	1.1	
CHF	27	0.8	51	6.2	<.001
CKD	400	12.0	288	35.0	<.001
Cancer	191	5.7	140	17.0	<.001
Diabetes	371	11.1	243	29.5	<.001
COPD	25	0.7	29	3.5	<.001
IBD	51	1.5	14	1.7	.718
BMI					
Underweight	99	3.0	12	1.5	<.001
Normal Weight	1085	32.4	192	23.3	
Overweight	1127	33.7	269	32.6	
Obesity	899	26.9	328	39.8	
Missing	134	4.0	23	2.8	

The cohort was 61.1% female with a mean age of 48 years (±20). In patients categorized as Normal RDW (*n* = 3344), 60.2% were female compared to 64.7% of patients with High RDW (*p* = .017). Mean age was significantly different between groups; the average age for Normal RDW patients was 46 years (±19) and for High RDW patients, 58 years (±22; *p* < .001). Among all patients hospitalized due to COVID-19 (*n* = 829), the mean time between positive PCR test and hospitalization was 21.3 (±37.4) days, whereas the median time was 5 [IQR 1–18] days. Other baseline characteristics were presented and compared between groups in Table 1, including SES, chronic diseases, and BMI.

**Figure 2.**
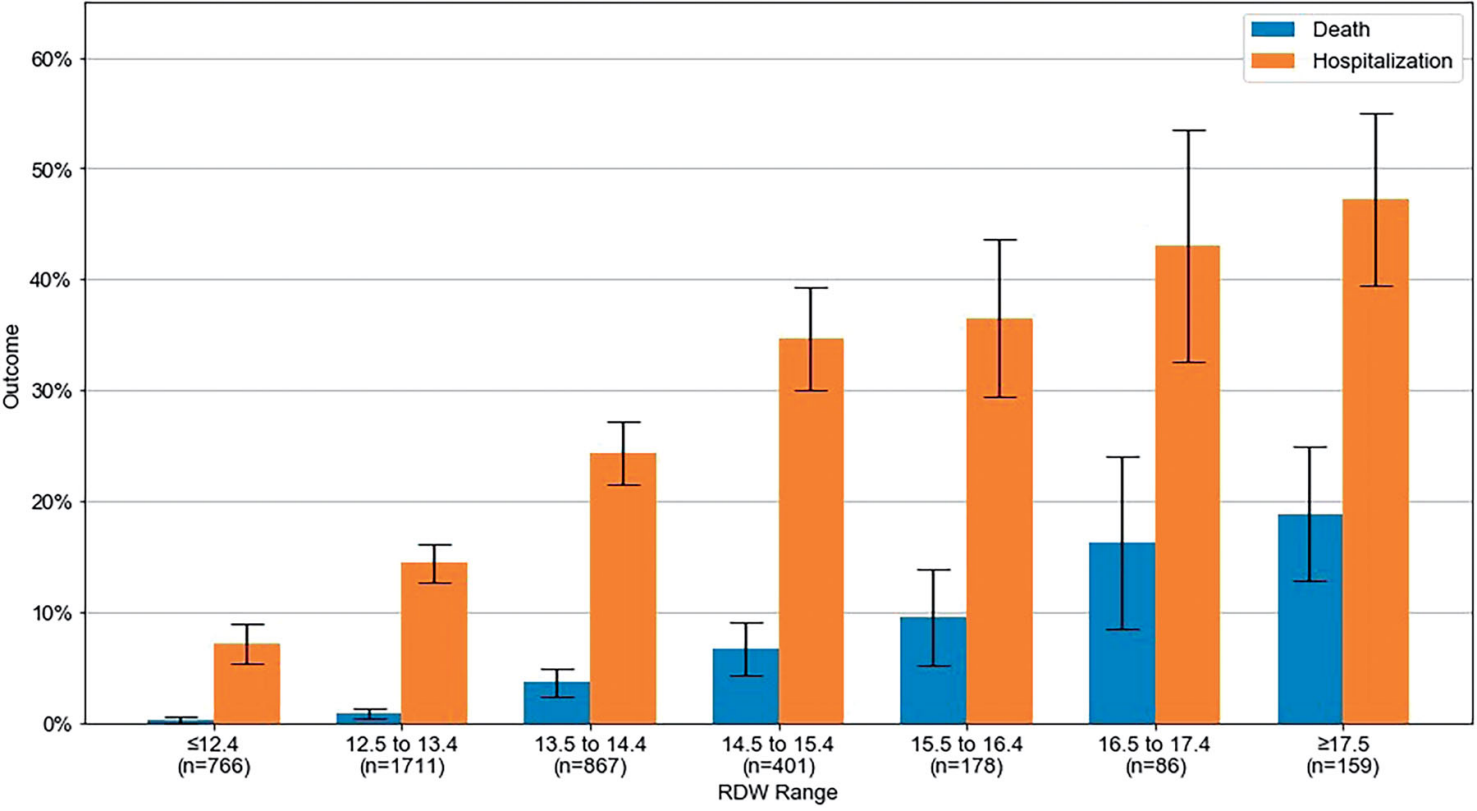
Death and hospitalization proportion per RDW % range with 95% CI.

The mortality and hospitalization proportions along various ranges of RDW percentage values are presented in Figure 2. Among patients with RDW values between 13.5 and 14.4, 24.3% (95% CI = 21.5–27.2) were hospitalized and 3.7% (95% CI = 2.4–4.9) died, whereas for RDW between 14.5 and 15.4, 34.7% (95% CI = 30.0–39.3) were hospitalized and 6.7% (95% CI = 4.3–9.2) died. Additionally, for RDW between 16.5 and 17.4, 43.0% (95% CI = 32.6–53.5) were hospitalized and 16.3% (95% CI = 8.5–24.1) died. In patients with RDW above or equal to 17.5, 47.0% (95% CI = 39.4–54.9) were hospitalized and 18.9% died (95% CI = 12.8–24.9). Time to mortality was also assessed *via* Kaplan Meier survival curves, where RDW ranges were presented as in Figure 2 (Supplementary Figure 1) and as quartile ranges (Supplementary Figure 2).

### Logistic regression models

The first multivariate logistic regression in this study evaluated hospitalization as the independent variable (outcome) with baseline RDW lab result (continuous variable), age, and sex as predictors in the model. Overall, 513 (15.3%) of Normal RDW patients and 316 (38.3%) of High RDW patients were hospitalized during follow-up. Results showed that RDW and age were significantly associated with a hospitalization outcome with an OR of 1.278 (95% CI = 1.222–1.336) and 1.028 (95% CI = 1.024–1.032), respectively. Sex was not significantly associated with the outcome (*p* = .292). In addition to the above predictors, comorbidities and BMI were added into a logistic regression (Table 2: Model 1). After adjusting for the additional covariates, RDW and age were still significantly associated with risk factors for hospitalization. Similar results were obtained when RDW was evaluated as a discrete variable, coded as Normal and High RDW (≥14.5), where RDW and age were significantly associated with hospitalization with ORs of 2.541 (95% CI = 2.124–3.039) and 1.029 (95% CI = 1.025–1.033), respectively. Sex was not significantly associated with hospitalization in this model (*p* = .217). High RDW and age were still significant when comorbidities and BMI were added in the regression (Table 2: Model 2).

**Table 2.** Logistic regression for hospitalization outcomes for COVID-19 patients with an RDW value up to two weeks before positive PCR test (*n* = 4168).

	OR for hospitalization	95% C.I. for OR		*p*-Value
		Lower	Upper	
Model 1				
RDW (continuous)*	1.242	1.187	1.300	<.001
Age	1.016	1.011	1.022	<.001
Sex (female)	1.120	0.943	1.330	.197
CHF	1.273	0.777	2.084	.338
CKD	1.858	1.474	2.344	<.001
Cancer	1.310	1.002	1.712	.049
Diabetes	1.288	1.032	1.608	.025
COPD	1.008	0.553	1.836	.980
IBD	0.723	0.341	1.530	.396
BMI (normal)	(Reference)			
BMI (underweight)	1.209	0.670	2.183	.528
BMI (overweight)	1.188	0.958	1.474	.117
BMI (obese)	1.273	1.024	1.583	.030
BMI (missing)	0.725	0.416	1.265	.258
Model 2				
High RDW (discrete)*	2.232	1.853	2.688	<.001
Age	1.017	1.012	1.022	<.001
Sex (female)	1.137	0.958	1.350	.141
CHF	1.397	0.857	2.278	.180
CKD	1.829	1.450	2.306	<.001
Cancer	1.340	1.026	1.749	.032
Diabetes	1.291	1.034	1.612	.024
COPD	0.963	0.527	1.759	.903
IBD	0.740	0.349	1.567	.431
BMI (normal)	(Reference)			
BMI (underweight)	1.274	0.717	2.264	.410
BMI (overweight)	1.194	0.964	1.480	.105
BMI (obese)	1.281	1.031	1.592	.025
BMI (missing)	0.709	0.406	1.236	.225

*Note that the first model assesses RDW as a continuous variable and the second model assesses RDW as a bivariate (High RDW compared to Normal RDW).

Sensitivity analyses were performed excluding patients hospitalized more than 28 days post-index date, where 13.5% Normal RDW patients and 36.7% High RDW patients had a hospitalization outcome between 0 and 28 days after positive PCR test. Logistic regression models had very similar results (Supplementary Table 2).

Similar analyses were performed for mortality as an outcome in place of hospitalization. Overall, 50 (1.5%) of patients with Normal RDW and 88 (10.7%) patients with High RDW died during follow-up. Before adjusting for comorbidities and BMI, RDW (continuous) was entered into the model with age and sex, where results showed that RDW and age were significantly associated with mortality, with ORs of 1.288 (95% CI = 1.195–1.388) and 1.116 (95% CI = 1.098–1.134), respectively. Once more, sex was not significantly associated in the model (*p* = .080). Similar results occurred with added comorbidities and BMI (Table 3: Model 3). When RDW was entered as a discrete variable in the model, sex was not significant (*p* = .062), but RDW and age were significantly associated with the outcome with ORs of 3.105 (95% CI = 2.101–4.590) and 1.113 (95% CI = 1.095–1.131), respectively. Alike results were obtained when adjusting for comorbidities and BMI in the regression (Table 3: Model 4).

**Table 3.** Logistic regression for mortality outcomes for COVID-19 patients with an RDW value up to two weeks before positive PCR test (*n* = 4168).

	OR for mortality	95% C.I. for OR		*p*-Value
		Lower	Upper	
Model 3				
RDW (continuous)*	1.263	1.166	1.368	<.001
Age	1.096	1.076	1.116	<.001
Sex (female)	0.682	0.456	1.019	.062
CHF	1.347	0.707	2.566	.364
CKD	2.668	1.592	4.470	<.001
Cancer	0.842	0.529	1.341	.470
Diabetes	1.206	0.796	1.827	.376
COPD	1.594	0.687	3.700	.278
IBD	2.614	0.631	10.821	.185
BMI (normal)	(Reference)			
BMI (underweight)	2.694	0.715	10.150	.143
BMI (overweight)	0.568	0.343	0.939	.028
BMI (obese)	0.528	0.314	0.889	.016
BMI (missing)	1.464	0.470	4.563	.511
Model 4				
High RDW (discrete)*	2.911	1.928	4.395	<.001
Age	1.094	1.075	1.114	<.001
Sex (femal*e* = 1)	0.665	0.445	0.995	.047
CHF	1.422	0.746	2.711	.284
CKD	2.580	1.542	4.318	<.001
Cancer	0.874	0.551	1.387	.567
Diabetes	1.185	0.78	1.80	.424
COPD	1.339	0.57	3.15	.504
IBD	2.604	0.59	11.41	.204
BMI (normal)	(Reference)			
BMI (underweight)	3.509	0.96	12.88	.059
BMI (overweight)	0.544	0.33	0.90	.018
BMI (obese)	0.497	0.30	0.84	.008
BMI (missing)	1.410	0.46	4.37	.552

*Note that the first model assesses RDW as a continuous variable and the second model assesses RDW as a bivariate (High RDW compared to Normal RDW).

A sub-analysis was performed to evaluate hospitalization outcomes with patients under 50 years old, without any comorbidities (*n* = 2203; mean age = 32 [±9], and 71.1% female). RDW lab result, as a continuous variable, was entered into the model with age and sex. Results showed a significant association with hospitalizations for RDW (OR = 1.212 [95% CI = 1.129–1.301]), age (OR = 0.985 [95% CI = 0.971–0.999]) and sex (OR = 2.297 [95% CI = 1.617–3.262]) in this model.

## Discussion

In this retrospective real-world data analysis, we discerned that elevated RDW values within two weeks before the diagnosis of COVID-19 were significantly associated with increased odds of both hospitalization and mortality due to COVID-19. After adjusting for cofounders (including age, sex, comorbidities, and BMI), a high RDW, equal to or greater than 14.5%, was associated with 2.23 times increased odds of hospitalization (*p* < .001) and 2.91 times increased odds of death (*p* < .001).

As noted above, previous research has indicated a significant elevation in RDW with increasing age, such that evaluating elevated RDW as a morbidity marker may be more effective in a younger population cohort [26,27]. For this reason, a sub-analysis was performed with patients under the age of 50, excluding those with COPD, CHF, CKD, cancer, diabetes, or IBD. These results suggested that RDW holds a significant predictive value in determining the odds of hospitalization in healthy, younger patients. In this subgroup, an RDW above 14.5% before diagnosis, was associated with a 21% greater odds of hospitalization (*p* < .001).

The results indicate that elevated pre-diagnosis RDW levels are an independent risk factor for disease severity in COVID-19 patients. These findings are in line with recent literature displaying that elevated RDW may represent a pro-inflammatory state in the body as well as dysregulated immune system response [28,29]. The underlying inflammatory disturbance that expresses itself, in part, as an elevated RDW, may allow for increased susceptibility to disease progression and severity. This may be a particularly influential measurement for COVID-19 patients; severe COVID-19 disease course is characterized by a heightened pro-inflammatory response leading to the cytokine storm that causes rapid deterioration and death due to the development of COVID-19 associated acute respiratory distress syndrome (ARDS) [5,30].

The mean and median time from positive PCR test to hospitalization for the cohort was 21.3 and 5.0 days, respectively. Knowing the pre-diagnosis RDW value at the time of the COVID-19 PCR test, considering this short median allows for a modified clinical intervention in the weeks leading up to potential hospitalization after the time of diagnosis. The results may facilitate a change in COVID-19 patient management and encourage screening high-risk patients; according to the RDW lab results, RDW cut-off value of 14.5% provides a strong sensitivity (63.8%) and specificity (81.7%) for predicting COVID-19 related mortality. This cut-off value corroborates the 14.5% RDW cut-off value given in other COVID-19 research articles [4,30,31].

According to the ROC curves evaluating the predictive value of lab tests in Figure 1, haemoglobin and RBC also display large areas under the curve. Furthermore, at low values of specificity, they have higher sensitivity than RDW for both hospitalization and mortality. At ∼50% sensitivity and above, RDW is the leading predictor. For clinical purposes, the determination of higher specificity is also relevant to capture the “true negative” patients (in this case, patients without hospitalization or death). Further research should be performed to assess haemoglobin and RBC as predictors, including RDW for comparison, as they may prove to be strong diagnostic tests for COVID-19 related hospitalization or death.

A recent publication on the association of anisocytosis with short-term mortality in COVID-19 patients evaluated a cohort of 282 patients [30]. In their study, RDW was categorized as four quartiles (<12.9, 12.9–13.6, 13.7–14.6, and >14.6%). Results from a Cox Regression analysis suggested that RDW > 14.6% was associated with a 45% relative increase in mortality compared to RDW < 12.9%. The researchers also highlight that their data suggest an RDW of 14.5% as an optimal cut-point (identified by using maximal Youden index [highest sensitivity plus specificity]), with 72% sensitivity and 81% specificity to predict short-term mortality (30-day mortality). Although their study employed a different statistical analysis, the results suggest that elevated RDW is a risk factor for death and is comparable to this study's finding proposing High RDW COVID-19 patients have 2.91 times increased odds of death. Furthermore, their proposed RDW threshold of 14.5% sensitivity and specificity was similar to this study's RDW data, which advises a 63.8% sensitivity and 81.7% specificity for mortality prediction.

From a clinical point of view, this research will help physicians triage patients based on recently acquired lab values. Physicians, both in the outpatient clinics and in the emergency department, will be able to more effectively predict newly diagnosed COVID-19 patients' clinical course based on RDW values available in their medical records. Our results suggest that elevated RDW values, above 14.5%, carry greater odds of hospitalization and mortality. This may allow physicians and hospital staff to better manage at-risk patients that require greater oversight and early intervention.

### Strengths and limitations

Some methodological limitations in this study should be considered. First, the threshold used to define High RDW (≥14.5) is not defined as a national or international “upper limit” for RDW. Further research can be performed in order to evaluate the various RDW ranges (similar to those presented in Figure 2) to assess if there is a cut-off value better suited as a stronger predictor for COVID-19 related hospitalization and mortality. In addition, the follow-up time for this cohort varies; patients have between 14 days to 11 months of follow-up. Therefore, some patients may not have had enough time for the infection to progress into a COVID-19 related hospitalization or death. That being said, the median time to hospitalization was 5 days and all relevant hospitalizations likely occurred.

However, this study also presents several strengths, such as the use of real-world data from a large state-mandated healthcare provider. The main strength is that lab data from community blood tests were acquired and analyzed, rather than inpatient blood tests during hospitalization. Specifically, when evaluating RDW, it is of interest to analyze results before disease or infection onset.

## Conclusions

Elevated RDW results from community lab tests before COVID-19 diagnosis are strong, significant predictors for greater odds of morbidity and mortality. Following COVID-19 diagnosis, patients' recent RDW test, if available, should be added to the risk stratification process.

## Supplementary Material

Supplemental Material

## Data Availability

Data sharing is not applicable in this research article due to privacy matters and therefore it was not approved by the IRB.
