# Supplementary material for: Evaluating red blood cell distribution width from community blood tests as a predictor of hospitalization and mortality in adults with SARS-CoV-2: a cohort study
Source: Ann Med. 2021 Aug 19;53(1):1410–8. doi: 10.1080/07853890.2021.1968484 (PMC8381942; doi:10.1080/07853890.2021.1968484)
Supplement: Supplemental Material [file IANN_A_1968484_SM2223.zip › Supplemental files/Supplementary material.docx]

**Evaluating red blood cell distribution width from community blood tests as a predictor of hospitalization and mortality in adults with SARS-CoV-2: A cohort study**

**Corresponding author:** Tamar Banon

Contact phone: +972-3-7462572

HaMared 27, Tel Aviv, Israel 68125

tamarbanon@gmail.com

**Supplementary Material (for approval)**

**Supplementary Table 1.** Optimal cut-point values per lab test for the ROC curves describing COVID-19 related hospitalization and mortality outcomes

| **Lab** | **Hospitalization** | | | |
| --- | --- | --- | --- | --- |
|  | Cut-point | Sensitivity | Specificity | AUC |
| RDW | 13.75 | 55.5% | 73.3% | 0.692 |
| MCH | 29.55 | 47.8% | 57.9% | 0.531 |
| MCV | 89.95 | 54.2% | 58.4% | 0.582 |
| Neutrophils | 4.02 | 57.2% | 62.0% | 0.632 |
| Hemoglobin | 12.65 | 57.9% | 66.3% | 0.584 |
| Lymphocytes | 1.83 | 56.3% | 57.1% | 0.659 |
| Platelets | 218.50 | 52.8% | 57.4% | 0.590 |
| RBC | 4.43 | 59.5% | 62.7% | 0.572 |
| **Lab** | **Mortality** | | | |
|  | Cut-point | Sensitivity | Specificity | AUC |
| RDW | 14.35 | 66.7% | 80.4% | 0.816 |
| MCH | 29.55 | 49.3% | 57.0% | 0.542 |
| MCV | 90.95 | 63.8% | 65.8% | 0.690 |
| Neutrophils | 4.77 | 60.6% | 75.2% | 0.712 |
| Hemoglobin | 12.25 | 71.7% | 71.6% | 0.649 |
| Lymphocytes | 1.56 | 56.9% | 70.2% | 0.764 |
| Platelets | 225.50 | 50.4% | 50.9% | 0.670 |
| RBC | 4.20 | 68.1% | 74.2% | 0.490 |

**Supplementary Table 2:** Sensitivity analyses—Logistic regressions for hospitalization outcomes between 0 and 28 days post positive PCR test for COVID-19 patients with an RDW value up to two weeks prior to positive PCR test (n=4,007)

| **Model S1** | OR for Hospitalization | 95% C.I.for OR | | p-value |
| --- | --- | --- | --- | --- |
|  |  | Lower | Upper |  |
| RDW *(continuous)** | 1.252 | 1.193 | 1.314 | <0.001 |
| Age | 1.026 | 1.020 | 1.032 | <0.001 |
| Sex (female) | 1.008 | 0.835 | 1.217 | 0.934 |
| CHF | 1.166 | 0.700 | 1.942 | 0.554 |
| CKD | 1.890 | 1.479 | 2.415 | <0.001 |
| Cancer | 1.229 | 0.926 | 1.631 | 0.152 |
| Diabetes | 1.237 | 0.978 | 1.564 | 0.076 |
| COPD | 1.044 | 0.565 | 1.929 | 0.890 |
| IBD | 0.825 | 0.355 | 1.917 | 0.654 |
| BMI (normal) | (reference) |  |  |  |
| BMI (underweight) | 1.456 | 0.746 | 2.843 | 0.271 |
| BMI (overweight) | 1.253 | 0.982 | 1.599 | 0.070 |
| BMI (obese) | 1.315 | 1.028 | 1.681 | 0.029 |
| BMI (missing) | 0.600 | 0.300 | 1.200 | 0.149 |
| **Model S2** | OR for Hospitalization | 95% C.I.for OR | | p-value |
|  |  | Lower | Upper |  |
| High RDW *(discrete)** | 2.372 | 1.936 | 2.905 | <0.001 |
| Age | 1.026 | 1.020 | 1.033 | <0.001 |
| Sex (female) | 1.022 | 0.847 | 1.233 | 0.820 |
| CHF | 1.299 | 0.783 | 2.155 | 0.310 |
| CKD | 1.852 | 1.449 | 2.367 | <0.001 |
| Cancer | 1.263 | 0.953 | 1.673 | 0.104 |
| Diabetes | 1.229 | 0.971 | 1.556 | 0.086 |
| COPD | 0.991 | 0.534 | 1.838 | 0.978 |
| IBD | 0.853 | 0.367 | 1.981 | 0.711 |
| BMI (normal) | (reference) |  |  |  |
| BMI (underweight) | 1.557 | 0.815 | 2.974 | 0.180 |
| BMI (overweight) | 1.255 | 0.985 | 1.600 | 0.066 |
| BMI (obese) | 1.315 | 1.029 | 1.681 | 0.029 |
| BMI (missing) | 0.583 | 0.292 | 1.165 | 0.126 |

*Note that the first model assesses RDW as a continuous variable and the second model assesses RDW as a bivariate (High RDW compared to Normal RDW)

**Supplementary Figure 1.** Kaplan Meier survival curves for time to mortality by ranges of RDW results

**Supplementary Figure 2.** Kaplan Meier survival curves for time to mortality by quartile ranges of RDW results
