# Supplementary figures and images for: Evaluating red blood cell distribution width from community blood tests as a predictor of hospitalization and mortality in adults with SARS-CoV-2: a cohort study
Source: Ann Med. 2021 Aug 19;53(1):1410–8. doi: 10.1080/07853890.2021.1968484 (PMC8381942; doi:10.1080/07853890.2021.1968484)

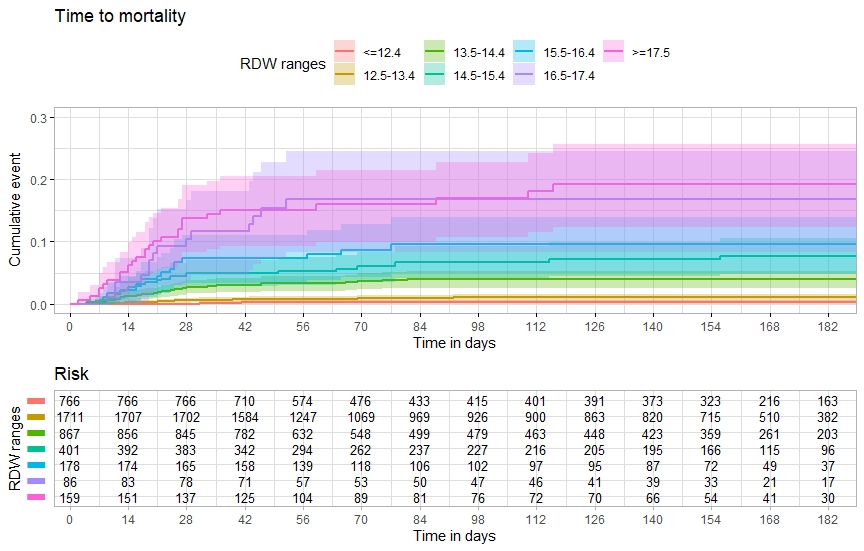

Supplement: Supplemental Material [file IANN_A_1968484_SM2223.zip › Supplemental files/Supplementary Figure 1.jpeg]

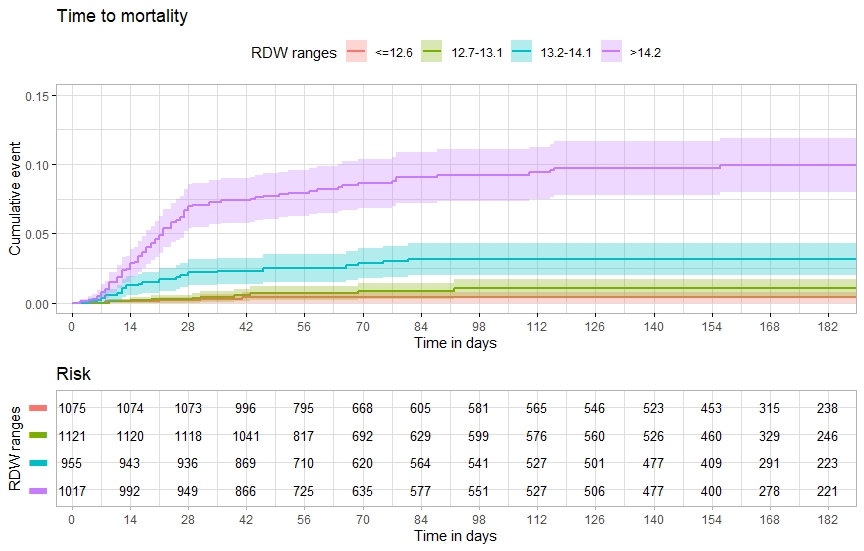

Supplement: Supplemental Material [file IANN_A_1968484_SM2223.zip › Supplemental files/Supplementary Figure 2.jpeg]
